# Transplant Trial Watch

**DOI:** 10.3389/ti.2023.11202

**Published:** 2023-03-21

**Authors:** John Matthew O’Callaghan

**Affiliations:** ^1^ University Hospitals Coventry and Warwickshire, Coventry, United Kingdom; ^2^ Centre for Evidence in Transplantation, University of Oxford, Oxford, United Kingdom

**Keywords:** randomised controlled trial, kidney transplant, vaccine, COVID-19, immune response

To keep the transplantation community informed about recently published level 1 evidence in organ transplantation ESOT and the Centre for Evidence in Transplantation have developed the Transplant Trial Watch. The Transplant Trial Watch is a monthly overview of 10 new randomised controlled trials (RCTs) and systematic reviews. This page of Transplant International offers commentaries on methodological issues and clinical implications on two articles of particular interest from the CET Transplant Trial Watch monthly selection. For all high quality evidence in solid organ transplantation, visit the Transplant Library: www.transplantlibrary.com.

Randomised Controlled Trial 1 Benefits of Switching Mycophenolic Acid to Sirolimus on Serological Response after a SARS-CoV-2 Booster Dose among Kidney Transplant Recipients: A Pilot Study.

*by Banjongjit, A., et al. Vaccines 2022; 10 (10): 09.*

## Aims

The aim of this study was to compare the immune response to the booster dose of BNT162b2 in renal transplant patients who remain on the standard immunosuppressive regimen [tacrolimus (TAC), MPA, and prednisolone] versus those who switch to the mammalian target of rapamycin inhibitor (mTORi), TAC, and prednisolone regimen.

## Interventions

Participants were randomised to either continue the standard regimen or switch to a sirolimus (an mTORi), TAC, and prednisolone regimen.

## Participants

28 kidney transplant recipients.

## Outcomes

The main outcomes of interest were change in anti-SARS-CoV-2 S antibody level pre- and post-BNT162b2 vaccination, and adverse events.

## Follow-Up

6 months.

## CET Conclusion

This is a very interesting pilot study on vaccine responses on different immunosuppressive regimens. For 2 weeks prior and 2 weeks after vaccination (ChAdOx-1), recipients were randomised to switch Mycophenolate to Sirolimus, or stay on usual immune suppression. The study was conducted in a single centre, and patients were randomly assigned by computer algorithm in an unblinded manner. Only 28 recipients met the inclusion criteria. Whilst the anti-SARS-CoV-2 S antibody levels increased significantly in both groups, the switching group had a significantly higher level in comparison. It is critical to consider adverse events, and in this study 2 patients in the sirolimus group experienced mouth ulcers that healed after returning to mycophenolate at the end of study. There were no significant changes in serum creatinine, urine albumin or any other significant symptoms. This study shows that a very short conversion window from mycophenolate to sirolimus can significantly improve vaccine antibody responses in kidney transplant recipients.

## Jadad Score

2.

## Data Analysis

Per protocol.

## Allocation Concealment

Yes.

## Trial Registration

TCTR20220404001.

## Funding Source

Non-industry funded.

Randomised Controlled Trial 2 Alternative Strategies to Increase the Immunogenicity of COVID-19 Vaccines in Kidney Transplant Recipients not Responding to Two or Three Doses of an mRNA Vaccine (RECOVAC): A Randomised Clinical Trial.

*by Kho, M. M. L., et al. The Lancet Infectious Diseases 2022 [record in progress].*

## Aims

This study aimed to compare the immunogenicity of a double dose vaccine, heterologous vaccination, and temporary discontinuation of mycophenolate mofetil or mycophenolic acid to that of a control single dose mRNA-1273 vaccination, in kidney transplant recipients who do not respond to two or three doses of an mRNA vaccine.

## Interventions

In the first cohort, participants were randomised to receive a single dose of mRNA-1273, two doses of mRNA-1273, or the Ad26.COV2-S vaccine. In the second cohort, patients receiving triple immunosuppressive therapy were randomised to either continue mycophenolate mofetil or mycophenolic acid, or discontinue mycophenolate mofetil or mycophenolic acid, from 1 week before until 1 week after being vaccinated with a single 100 μg dose of mRNA-1273.

## Participants

230 kidney transplant recipients were randomised in the first cohort and 103 kidney transplant recipients were randomised in the second cohort.

## Outcomes

The primary endpoint was the percentage of participants with a spike protein (S1)-specific IgG concentration ≥10 BAU/mL 28 days following vaccination. Secondary endpoints included the presence of virus neutralising antibodies, serum concentration of S1-specific IgG, and SARS-CoV-2 specific T-cell response and safety.

## Follow-Up

28 days.

## CET Conclusions

This is another very interesting study on vaccine responses in kidney transplant recipients. In this complex study kidney transplant recipients were randomised to receive either an mRNA vaccine of 100 or 200 μg (mRNA-1273) versus a viral vector vaccine (Ad26.COV2-s). A small group was also randomised to continue or discontinue mycophenolate for 1 week before and 1 week after. The study showed again, as has been seen elsewhere, that a significant proportion of transplant recipients do not seroconvert after two or even three doses of SARS-CoV-2 vaccine (34% and 20%). Vaccination with 200 μg mRNA vaccine was not significantly better than 100 μg or the viral vector vaccine. Stopping mycophenolate for 1 week and before and 1 week after did not have any significant impact on vaccine response either. The study was adequately randomised and powered, however it was not blinded. Given the objective nature of the results this is not of significant concern for systematic bias in the reporting of the results. The study was funded by The Netherlands Organization for Health Research and Development and the Dutch Kidney Foundation.

## Jadad Score

3.

## Data Analysis

Per protocol.

## Allocation Concealment

Yes.

## Trial Registration

ClinicalTrials.gov—NCT05030974.

## Funding Source

Non-industry funded.

## Clinical Impact Summary

For this month’s clinical impact summary we have selected two related clinical trials. Transplant recipients do not have the same initial response to COVID-19 vaccines as other members of the population, leaving them at increased risk. Hence a strategy to improve vaccine response is critical.

The first study by Bangonjit et al., from Bangkok, Thailand is a relatively small pilot study from a single centre. Transplant patients in this study had previously received two doses of ChAdOx-1 vaccine (viral vector) and one dose of BNT162b2 (mRNA) vaccine (*n* = 28). Patients received a booster dose of BNT162b2 vaccine, but were randomised to switch from mycophenolic acid to sirolimus for 2 weeks prior and up to 2 weeks post-vaccination. The COVID-19 antibody levels post-vaccination were significantly higher in the sirolimus group than the mycophenolate group, without a significant number of adverse events. However, the study was very small and hence less common, although potentially very severe, events related to switching immune suppression may not have been revealed. There was only one seronegative patient, who remained seronegative after the booster dose.

The results from this study support those found by the team from the OPTIMIZE trial in the Netherlands, published earlier this year (1). Although there are some slight differences in patient group and immune suppression. They also echo the results of another study also published earlier this year (2).

The second study this month, by Kho et al, is from four centres in Netherlands, and includes 345 patients, although the study was complex with multiple subdivisions and randomisations. Kidney transplant recipients who remained seronegative after two, or three, doses of mRNA vaccine were included. Patients were randomised to receive an mRNA vaccine of 100 or 200 μg (mRNA-1273) versus a viral vector vaccine (Ad26.COV2-s). In addition, a small group receiving 100 μg mRNA vaccine was also randomised to continue or discontinue mycophenolate for 1 week before until 1 week after the third vaccine dose (*n* = 108).

A significant proportion of these recipients still did not seroconvert after two or even three doses of SARS-CoV-2 vaccine (34% and 20%). There was no significant difference in seroconversion rate comparing 200 μg mRNA vaccine to 100 μg, or the viral vector vaccine. Stopping mycophenolate for 1 week before and 1 week after did not have any significant impact on vaccine response either.

This study highlights the need for the third and even fourth COVID-19 booster vaccines to improve seroconversion in transplant recipients. Whilst this second study did not show an improved vaccine response when stopping mycophenolate, it was for a relatively short period only. Stopping mycophenolate for a longer period may be necessary to improve the immune response, however it may require the addition of another immune suppressant (such as sirolimus) to therapy during the switch period. Published studies assessing this concept have so far been small and therefore not reliable in assessing safety.

## References

[1] de Boer SE Berger SP van Leer-Buter CC Kroesen BJ van Baarle D Sanders JF Enhanced Humoral Immune Response after COVID-19 Vaccination in Elderly Kidney Transplant Recipients on Everolimus versus Mycophenolate Mofetil-Containing Immunosuppressive Regimens. J F Transplant (2022) 106(8):1615–21. 10.1097/TP.0000000000004177 PMC9311282 35546527

[2] Schrezenmeier E Rincon-Arevalo H Jens A Stefanski AL Hammett C Osmanodja B Temporary Antimetabolite Treatment Hold Boosts SARS-CoV-2 Vaccination-specific Humoral and Cellular Immunity in Kidney Transplant Recipients. JCI Insight (2022) 7(9):e157836. 10.1172/jci.insight.157836 35349490 PMC9090237

